# Exploring the Complex Link between Autophagy, Regulated Cell Death, and Cell Fate Pathways in Cancer Pathogenesis and Therapy

**DOI:** 10.3390/cells12030498

**Published:** 2023-02-03

**Authors:** Mohammad Amin Moosavi, Mojgan Djavaheri-Mergny

**Affiliations:** 1 Department of Molecular Medicine, Institute of Medical Biotechnology, National Institute of Genetic Engineering and Biotechnology, Tehran P.O. Box 14965/161, Iran; 2 Centre de Recherche des Cordeliers, INSERM UMRS 1138, Sorbonne Université, Université de Paris, Equipe 11 Labellisée par la Ligue Contre le Cancer, 75006 Paris, France; 3 Metabolomics and Cell Biology Platforms, Institut Gustave Roussy, 94805 Villejuif, France

Autophagy is a catabolic lysosomal-dependent pathway involved in the degradation of cellular materials, supplying precursor compounds and energy for macromolecule synthesis and metabolic needs [1,2]. There are three distinct forms of autophagy, including macroautophagy (simply referred to here as autophagy), microautophagy and chaperone-mediated autophagy (CMA) [1]. All three are essential in regulating several key biological processes including cell death, metabolism, stress responses, as well as immune surveillance and disease development [2,3]. At early stages of tumorigenesis, autophagy is reduced, favoring cancer formation by increasing DNA damage. However, at later stages and in established tumor cells, autophagy is usually reactivated as an adaptive response to stressful conditions, helping to sustain cancer stem cells (CSCs) and their tumorigenic potential and contributing overall to resistance to cancer treatments [4]. As autophagy has both positive and negative roles in cancer, both its inhibition and activation may have therapeutic benefits for cancer patients.

Despite making much progress in understanding the molecular mechanisms of autophagy in cancer, many questions remain concerning its role in cancer development and treatment. This Special Issue of *Cells*, entitled “molecular connections between autophagy, programmed cell death pathways and differentiation in cancer cells”, presents some of the latest findings on the role of autophagy in regulated cell death pathways, metabolism, and immune responses, in addition to showcasing its involvement in cancer development, the maintenance of CSCs and resistance to cancer therapy. This issue features six review articles and eight original studies.

The role of autophagy in three different cancer types has been summarized in three comprehensive review articles. The first, submitted by Gabriela Reyes-Castellanos et al., is a comprehensive overview of the contribution of autophagy to metabolic reprogramming and therapeutic resistance in pancreatic tumors [5]. This review highlights four major aspects: key features of pancreatic ductal adenocarcinoma (PDAC), the role of autophagy in tumor metabolism, therapeutic approaches using autophagy inhibitors in PDAC, and autophagy-based strategies to overcome resistance in PDAC. The authors identify several key elements that link autophagy to PDAC, such as genetic alterations, mitochondrial metabolism, the tumor microenvironment (TME), and the immune system. They suggest that a combination therapy utilizing autophagy inhibitors alongside chemotherapy and/or drugs targeting either metabolic pathways or the immune response may be beneficial.

The second review article by Estelle Espinos et al. [6] provides an in-depth examination of the dual roles of autophagy (i.e., pro-survival or pro-death) in response to crizotinib treatment in ALK (anaplastic lymphoma kinase) positive anaplastic large cell lymphoma (ALCL). The authors explore several potential mechanisms that underlie the pro-survival effects of autophagy, such as the inhibition of apoptosis and the maintenance of cancer stemness. In contrast, they suggest that the pro-death effects of autophagy may be a result of the removal of pro-survival substrates, the formation of platforms for cell death complexes, or the re-activation of the anti-tumor immune responses. Finally, this review discusses how the modulation of autophagy might lead to the better management of ALK positive ALCL patients.

The third review by Nicolas J. Niklaus et al. focuses on the regulation and role of autophagy during normal breast development, breast carcinogenesis, breast cancer dormancy as well as breast CSCs [7]. The authors shed light onto the potential roles of autophagy in the traits of aggressive breast cancer cells such as migration, invasion, and therapeutic resistance. In conclusion, the authors emphasize the multifaceted role of autophagy in breast cancer, a function which depends on the biology of the breast cancer subtype, stage, and prior treatment course. Moreover, the authors underscore the importance of conducting research into autophagy biomarkers in order to determine the effects of early versus delayed autophagy modulation in personalized breast cancer care.

The multifaced role of autophagy has been also reviewed by Ali Zahedi-Amiri et al. in the context of oncolytic virotherapy [8]. Oncolytic viruses (OV) are defined as viruses that preferentially infect and lyse cancer cells. Depending on the cell type and oncovirus, autophagy may either promote oncolysis by inducing of immunogenic cell death or act as a defense mechanism to limit OV cytotoxicity. In the same vein, the authors suggest that autophagy can either activate or suppress inflammatory responses in the tumor microenvironment, a capacity that varies depending on the type of immune cell and its context. Finally, they discuss the potential of modulating autophagy in combination of oncolytic as an approach to cancer immunotherapy.

This Special Issue includes a review by Emma Cosialls et al. on the topic of ferroptosis, a regulated cell death pathway that depends on iron [9]. The authors discuss the importance of iron metabolism for the maintenance of cancer stem cells, a subpopulation of tumor cells that can drive tumor development and growth and generally can cause relapses. Furthermore, the review offers an in-depth analysis of ferroptosis and its regulation by autophagy. The authors present the involvement of autophagy in the formation and control of ferroptosis in detail. Finally, they discuss the potential to exploit the manipulation of iron accumulation through the induction of ferroptosis to target CSCs.

Growing evidence suggests that components of autophagy machinery have a role in processes beyond autophagy. In the original article by Kelly Airiau et al. this is explored by examining the role of autophagy regulatory proteins in calcium signaling and apoptosis in NB4 acute promyelocytic leukemia (APL) cells upon tumor necrosis factor-related apoptosis-inducing ligand (TRAIL) treatment [10]. The authors show that ATG7 and p62/SQSMT1 both regulate TRAIL-induced calcium influx and apoptosis when they are recruited to the death-inducing signaling complex (DISC). Furthermore, administering *all*-trans retinoic acid (ATRA) prior to the use of TRAIL enhances apoptosis in NB4 cells by increasing the recruitment of p62/SQSMT1 and ATG7 to the DISC. This suggests that the combination of ATRA and TRAIL may be particularly effective for the treatment of APL.

A further molecular connection between autophagy and calcium signaling was identified by Faten Merhi et al. in their study [11]. The authors demonstrate that ATRA induces the activation of autophagy and AMP-activated protein kinase (AMPK) in APL cells via the stimulation of store-operated calcium (SOC) channels and calmodulin-dependent protein kinase kinase 2 (CAMKK2), respectively, thereby establishing a link between calcium signaling and autophagy. Moreover, they demonstrate that the pharmacological inhibition of SOC channels and CAMKK2 enhances ATRA-induced cell death and differentiation. This study not only reveals the SOC channels and CAMKK2 axes to be novel pathways regulated by ATRA, but also identifies these two pathways as potential therapeutic targets to augment the anti-cancer effects of ATRA in APL patients.

Sanaz Dastghaib et al. investigate the interaction between autophagy and unfolded protein response (UPR), an adaptive response triggered by endoplasmic reticulum (ER) stress, in sensitizing glioblastoma (GBM) cells to chemotherapy-induced cell death [12]. The authors provide evidence that simvastatin (Simva) enhances temozolomide (TMZ)-induced cell death in GBM cells through UPR-mediated autophagy flux in U251 (p53 mutant) and U87 (p53 wild type) GBM cells. They discuss that the p53 status has a critical role in determining UPR and autophagy outcomes in Simva–TMZ-treated GBM cells.

The research paper of Eun-Hye Jang et al. makes strides in improving our understanding of the cytotoxic mechanism of valproic acid (VPA) in the SH-SY5Y neuroblastoma cell line [13]. VPA, a branched fatty acid, is known to induce mitochondrial dysfunction. The authors have uncovered a role of MTOR-dependent autophagy flux in the control of cell death of SH-SY5Y cells, with increased levels of LC3B-II levels accompanied by the decreased levels of p62/SQSMT1 expression. Additionally, FOXO3a was found to be localized in the nucleus during VPA-induced cell death.

The research article of Alexandra Nguyen et al. broadened our understanding on the mechanism of hydroxyurea, a suppressor of de novo dNTP synthesis, and entinostat, as a class 1 inhibitor of epigenetic modifiers of the histone deacetylase (HDAC) [14]. The results demonstrated that entinostat promotes the induction of DNA replication stress by hydroxyurea in primary murine PDAC cells, thereby providing new insights into HDAC-dependent replication stress and checkpoint kinase Ataxia Telangiectasia Mutated (ATM)-dependent pro-apoptotic programs in PDAC cells.

Cardiac glycosides (CGs) are compounds used to treat heart failure and arrhythmia that have recently been identified as potential anticancer drugs. Jan Škubník,Vladimíra et al. present a comprehensive review of the current knowledge of the anticancer activity of these cardiac glycosides as modulators of autophagy [15]. The authors point out that the effects of CGs on autophagy can vary depending on the cell type and specific CG. By inhibiting the Na^+^/K^+^-ATPase (NKA), CGs can protect cells against autosis, a form of cell death that is regulated by autophagy and Na^+^/K^+^-ATPase. On the other hand, CGs may contribute to autosis by causing an ion imbalance and other as-yet-unknown mechanisms. Further research is necessary to better understand the anticancer effects of CGs, and the authors suggest that the combination of CGs could be an effective strategy for cancer treatment.

In the same area, Dawei Liu et al. discuss how autophagy inducers could be used as cardioprotective drugs for the treatment of cancer patients at risk of cardiac disease [16]. Through high-throughput screening, the authors identified six molecules—two cardiac glycosides (digitoxigenin and digoxin), minaprine, SG6163F, VP331, and LOPA87—that could prevent necrotic and apoptotic cell death in rat cardiomyoblasts and rat neonatal ventricular myocytes. Mechanistically, these compounds altered autophagic proteins BECN1 and ATG5, modified mitochondrial dynamics and/or biogenesis and stimulated ATP production by increasing both aerobic and anaerobic metabolism. The authors suggested that digitoxigenin and digoxin (which are already FDA-approved drugs) could be repositioned and combined with radiotherapy or chemotherapy for further preclinical evaluation.

Tereza Losmanova et al. address the role of CMA, a less explored subtype of autophagy, in advanced non-small cell lung cancer (NSCLC) after neoadjuvant therapy [17]. Although the immunohistochemical expression of two CMA markers, LAMP2A and HSPA8 was not associated with tumor response or neoadjuvant therapy, high levels of LAMP2A were associated with longer overall survival and disease-free survival in patients with locally advanced NSCLC. These results suggest that offering LAMP2A could be used as an independent prognostic biomarker candidate for NSCLC.

Pawan Sharma et al. conduct an observational study to investigate the crosstalk between UPR, autophagy and apoptosis and their effects on lung function in patients with idiopathic pulmonary fibrosis (IPF) [18]. They find increased protein levels of UPR (BiP and XBP1) and apoptosis (cleaved CASPASE 3) markers to be associated with decreased lung function, while increased autophagy marker (LC3B-II) is seen to be significantly associated with increased diffusion capacity. The authors conclude that, in the context of active UPR, changes in autophagy flux may play an important role in determining lung function. This study has potential clinical relevance, as the evaluation of UPR, autophagy and apoptosis markers could be used both therapeutically and diagnostically in IPF treatment.
